# Efficacy of Er:YAG Laser as a Debridement Method in Surgical Treatment for Peri-Implantitis: A Systematic Review

**DOI:** 10.3290/j.ohpd.b3818041

**Published:** 2023-01-18

**Authors:** Lei Li, Yingqi Liu, Jiaojiao Guo, Xueqiang Zhang, Zhihai Jin, Jing Zhang

**Affiliations:** a Prosthodontist, Department of Stomatology, HanDan Central Hospital, Hebei Handan, China. Idea, hypothesis, experimental design, performed the experiments, wrote the manuscript, proofread the manuscript, performed statistical evaluation.; b Professor, Department of Stomatology, HanDan Central Hospital, Hebei Handan, China. Performed the experiments and proofread the manuscript.; c Periodontist, Department of Stomatology, HanDan Central Hospital, Hebei Handan, China. Performed the experiments, proofread the manuscript.; d Professor, Department of Stomatology, HanDan Central Hospital, Hebei Handan, China. Experimental design, contributed to discussion.; e PhD Student, Department of Orthopedics, HanDan First Hospital, Hebei Handan, China. Performed statistical evaluation, proofread the manuscript.; f Dentist, Department of Stomatology, HanDan Central Hospital, Hebei Handan, China. Performed statistical evaluation and proofread the manuscript.

**Keywords:** efficacy, Er:YAG laser, peri-implantitis, systematic review

## Abstract

**Purpose:**

The present study systematically reviewed randomised controlled trials (RCT) to investigate the efficacy of Er:YAG laser (ERL) as a debridement method in surgical treatment of advanced peri-implantitis.

**Materials and Methods:**

An electronic database search and a manual search were performed until March 2022. Outcome measures were clinical attachment level (CAL) gain, probing depth (PD) reduction, plaque index (PI) and bleeding on probing (BOP). The addressed PICO question was: Is ERL an effective debridement tool in the surgical treatment of advanced peri-implantitis?

**Results:**

Five eligible randomised clinical trials (RCTs) were included in the qualitative analysis, one of which had unclear risk of bias. One study reported a statistically significant difference in terms of implant CAL gain and PD reduction in favour of the experimental group vs the control group, while four studies did not report any difference between the two groups.

**Conclusion:**

Due to methodological heterogeneity, such as non-standard control groups and laser parameters, this systematic review demonstrated inconclusive findings in terms of the efficacy of Er:YAG laser as a debridement method in surgical treatment of advanced peri-implantitis. The results of this review should be considered preliminary and further, well-designed studies with standardised comparators with laser parameters are warranted.

Dental implants are currently an ideal choice for people who have lost their teeth for various reasons. However, biological complications frequently occur.^32^ Such biological complications include an inflammatory response in the soft tissues and bone surrounding the implants.The inflammatory lesions located in the soft tissues are defined as peri-implant mucositis and are reversible.^22^ If the inflammatory lesions are allowed to advance, loss of bone beyond the crestal bone level will occur, which is irreversible.^3^ Such condition is defined as peri-implantitis.^14^ A diagnosis of peri-implantitis requires: (i) bleeding and/or suppuration on gentle probing; (ii) an increased probing depth compared with previous examinations; and (iii) bone loss beyond crestal bone level, changes resulting from initial bone remodelling.^21^ The literature reports that the prevalence of peri-implantitis approximately is 28% to 77% of the subjects as well as 12% to 43% of the implant sites.^12^

Peri-implantitis is an inflammatory process, with numerous risk factors leading to its occurrence and development.^4,9^ Such risk factors include: history of periodontal disease, diabetes, smoking, plaque control or poor oral hygiene, crown contour, excess adhesive cement subgingivally around the implant, and metal corrosion or release of ions from metallic implants.^5,10^ The primary cause of peri-implant disease is the colonisation of plaque microorganisms on the implant surface.^33^ Therefore, the treatment method mainly removes the plaque biofilm on the surface of the implant. Treatment methods include surgical and non-surgical methods.^23^ Non-surgical mechanical debridement instruments include power-driven air-polishing devices, Er:YAG lasers, metal (e.g. titanium) curettes, and ultrasonic curettes with plastic sleeves. Non-surgical therapy is always the first-choice intervention, and in combination with proper oral hygiene, is useful in treating peri-implant mucositis. It usually provides clinical improvements of peri-implantitis, but may be insufficient to treat advanced cases. If the disease remains after non-surgical therapy, surgical interventions should be considered. Commonly used surgical methods include access surgery, resective surgery, or a regenerative procedure(e.g. guided bone regeneration [GBR]).^21^ Recent systematic review results show that Er:YAG laser has attracted much attention in the treatment of peri-implant diseases.^1,18,32^ However, the literature reports conflicting results. Although several studies found Er:YAG laser to have a bactericidal function and disinfect the contaminated implant surface without damaging it,^20,24,31^ other studies have shown the presence of micro-cracks and signs of coagulation, melting, and microfractures.^25^ As such, the principal objective of the present systematic review was to explore the efficacy of Er:YAG laser as a debridement method in surgical treatment of advanced peri-implantitis.

## Materials and Methods

### Focused Question

The Preferred Reporting Items for Systematic Review and Meta Analysis (PRISMA) guidelines were followed^13^ and a focused question was developed. The addressed PICO question was: Is ERL effective as a debridement method in the surgical treatment of advance peri-implantitis?

### Selection Criteria

Studies that did not fulfill the inclusion criteria were excluded. The inclusion criteria of the present review followed the PICOS question: (Population) the patients in the included studies had to be diagnosed with peri-implantitis, with the disease persisting after non-surgicl therapy; (Interventions) the experimental group in the included studies had to involve Er:YAG laser therapy as a debridement method in surgical therapy; (Comparisons) the control group involved surgical treatment with MD (mechnical debridement or other conventional non-surgical debridement method rather than ERL); (Outcomes) the primary outcome measure was clinical attachment level (CAL) gain and secondary outcome measures were probing depth (PD) reduction, plaque index(PI) and bleeding on probing (BOP), with a minimum follow-up assessment at 6 weeks; and (Study design) the review was restricted to randomised clinical trials (RCTs) published in the English or Chinese language. Animal studies, in-vitro studies, opinion articles, letters to the editor, review articles, interviews, updates, abstract, and unpublished studies were excluded.

### Search Strategy

Five electronic databases were searched up to March 2022: PubMed, Cochrane Central Register of Controlled Trial,China National Knowledge Infrastructure (CNKI), Chinese Technical Periodicals VIP Database and WanFang. The literature search was conducted using the combinations of the following Medical Subject Heading (MeSH) and text words:((“peri-implantitis” OR “peri-implant” OR “peri-implants” OR “periimplant” OR “periimplants”) AND (“laser” OR “lasers” OR “laser therapy” OR “laser therapies” OR “erbium”)) NOT (“letter” OR “comment” OR “editorial”). In addition, a manual search was conducted and potentially relevant references were included.

### Screening Methods and Data Abstraction

Two reviewers (LL and YL) performed the search independently. Once the duplicates had been removed, titles and abstracts of all identified studies were screened for eligibility. Any disagreement between the two reviewers was resolved through discussion until consensus was reached, or through arbitration by a third examiner (XZ). The level of agreement was calculated using the k-score according to the criteria of Landis and Koch.^11^ Data were extracted from the included studies according to the following parameters: author/year, country, study design, subjects (sample size, mean age and male to female ratio), study groups, smoking status, mean outcomes, follow-up, and risk of bias.

### Risk of Bias in Individual Studies

The reviewers independently assessed the risk of bias for all the selected studies according to the Cochrane Handbook for Systematic Reviews of Interventions.^8^ Each criterion was classified as “high”, “low”, or “unclear” risk of bias. Five main fields (randomisation, allocation concealment, participants and professionals blinded to the study, blinding of outcome assessment, and other bias) were considered to assess the quality of the studies. Overall, studies were considered as: low risk of bias if all criteria were met; unclear risk of bias if one or more criteria were partly met; or high risk of bias if one or more criteria were not met.

### Statistical Analysis

No meta-analysis could be performed due to the methodological heterogeneity of the included studies, for example, study groups, laser/photosensitiser parameters, and a variation in the outcomes of periimplant parameters. Therefore, the outcomes are reported as a narrative review.

## Results

### Study Selection

**Fig 1:**
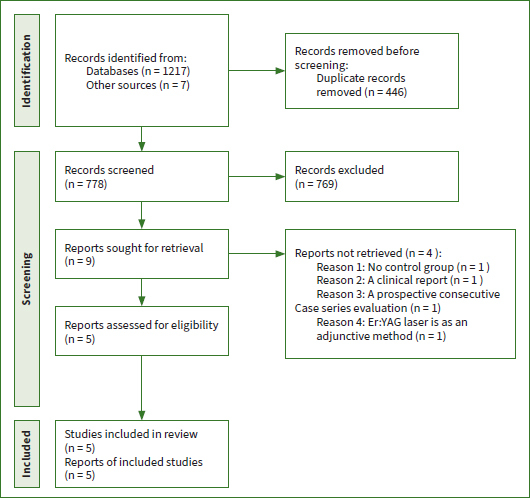
Flow chart of literature search.

A total of 1224 relevant titles and abstracts were identified according to the search strategy. Among those identified, 778 articles were included based on the title and abstract, after removing duplicates. Then, the titles and abstracts were screened by means of NoteExpress, and 769 articles were excluded according to the inclusion and exclusion criteria. After carefully and thoroughly reading the full text of the remaining nine articles, four articles were excluded because the inclusion criteria were not fulfilled (Table 1): one article did not have a control group, one article was a case report, one article was a prospective consecutive case series, and in one article, ERL was used as an adjunctive method. Overall, five studies^17,26-29^ met the inclusion criteria and were included in the present systematic review. The flow diagram of the study selection process is shown in Fig 1.

**Table 1:** Excluded clinical studies at the second stage of selection and the reason for exclusion

Publication (year)	Reason for exclusion
Lin et al (2019)	Case report
Clem et al (2019)	Prospective consecutive case series evaluation
Yamamoto et al (2021)	No control group
Wang et al (2021)	Er:YAG laser was used as an adjunctive method

### Study Characteristics

As shown in Table 2, all the studies were RCTs published between 2011 and 2021,^17,26-29^ four^26-29^ of which were written in English, and one^17^ was written in Chinese. The trials originated from Germany and China. In all studies, the number of patients ranged between 15 and 32, with the mean age ranging from 41 to 63 years. The male:female ratio was 42:71 in all the included studies. History of smoking was present in four studies. Four studies were designed for comparison between GBR+ERL and GBR+CPS (plastic curettes + cotton pellets + sterile saline),^26-29^ while one study was designed for comparison between GBR+ERL and GBR+MD.^17^ All studies reports the primary outcome measure “CAL gain” and secondary outcome measures “PD reduction” and “PI”. Only one study^17^ did not report the outcomes measure “BOP”. The follow-up period ranged from 6 months to 85 months.

**Table 2:** General description of included studies

Author, year	Country	Study design	Subject			Study groups		Smoker	Mean outcome (SD)	Follow-up (months)	Risk of bias
			Sample size	Gender ratio (M:F)	Mean age (years)	Test	Control				
Lu et al 2017	China	RCT	Patients: 25 Impants: 26 (test/control:14/12)	13:12	41.0 ± 4.3	GBR+ERL	GBR+MD	Non-smoker	CAL (mm) Test: 3.2 ± 3.3 Control: .2 ± 2.10 PD (mm) Test: 1.6 ± 1.53 Control:1.0 ± 0.95 PI Test:0.3 ± 0.3 Control:0.4 ± 0.39	6	High
Schwarz et al 2011	Germany	RCT	Patients: 32 (test/control: 16/16) Implants: 35 (test/control: 19/16)	11:21	Mean age 60.8 ± 10.9	GBR+ERL	GBR+CPS	Non-smoker or light smoker (< 10 cigarettes per day)	CAL (mm) Test: 1.5 ± 1.4 Control: 2.2 ± 1.4 PD Test:1.7 ± 1.4 Control:2.4 ± 1.5 PI Test: 0.4 ± 0.5 Control: 0.5 ± 0.6 BOP(%) Test: 47.8 ± 35.5 Control: 55.0 ± 31.1	6	Unclear
Schwarz et al 2012	Germany	RCT	Patients: 24 (test/control:10/14) Implants: 26 (test/control: 12/14)	8:16	Mean age 62.3 ± 10.0	GBR+ERL	GBR+CPS	Non-smoker or light smoker (< 10 cigarettes per day)	CAL (mm) Test: 1.0 ± 2.2 Control:1.2 ± 2.2 PD (mm) Test: 1.1 ± 2.2 Control: 1.5 ± 2.0 PI Test:0.2 ± 0.6 Control:0.0 ± 0.8 BOP(%) Test: 75.0 ± 32.6 Control: 54.9 ± 30.	24	High
Schwarz et al 2013	Germany	RCT	Patients: 17 (test/control: 7/10) Implants: 21 (test/control: 9/12)	6:11	Mean age 62.2 ± 0.0	GBR+ERL	GBR+CPS	Non-smoker or light smoker (< 10 cigarettes per day)	CAL (mm) Test: 1.2 ± 2.0 Control: 1.5 ± 2.0 PD (mm) Test: 1.3 ± 1.8 Control: 1.2 ± 1.9 PI Test: 0.4 ± 0.7 Control: 0.0 ± 1.1 BOP(%) Test:7 1.6 ± 24.9 Control: 85.2 ± 16.4	48	High
Schwarz et al 2017	Germany	RCT	Patients: 15 (test/control: 6/9 Implants: 15 (test/control: 6/9)	4 :11	Median age: 63	GBR+ERL	GBR+CPS	Non-smoker or light smoker (< 10 cigarettes per day)	CAL (mm) Test: 2.06 ± 2.52 Control: 2.76 ± 1.92 PD (mm) Test: 0.74 ± 1.89 Control: 2.55 ± 1.67 PI Test: -0.12 ± 0.60, Control: 0.17 ± 0.97 BOP(%) Test: 86.66 ± 18.26 Control: 89.99 ± 11.65	Test: 85.4 ± 3.36 Control: 83.8 ± 6.14	High

M: male; F: female; ERL: Er:YAG laser; MD: mechnical debridement; CPS: plastic curettes + cotton pellets + sterile saline.

**Table 3:** Laser and photosensitizer parameters of included studies

Author, Year	Er:YAG Laser brand	Wavelength (nm)	Energy fluence (J/cm^2^)	Power (W)	Irradiation time (s)	Optic fiber diameter (mm)	Number of laser sessions
Lu et al 2017	Fotona; Ljublana, Slovenia	2940	NA	1.2	NA	NA	NA
Schwarz et al 2011	elexxion delos; Radolfzell, Germany	2940	11.4	1	NA	NA	1
Schwarz et al 2012	elexxion delos	2940	11.4	1	NA	NA	1
Schwarz et al 2013	elexxion delos	2940	11.4	1	NA	NA	1
Schwarz et al 2017	elexxion delos	2940	11.4	1	NA	NA	1

**Fig 2:**
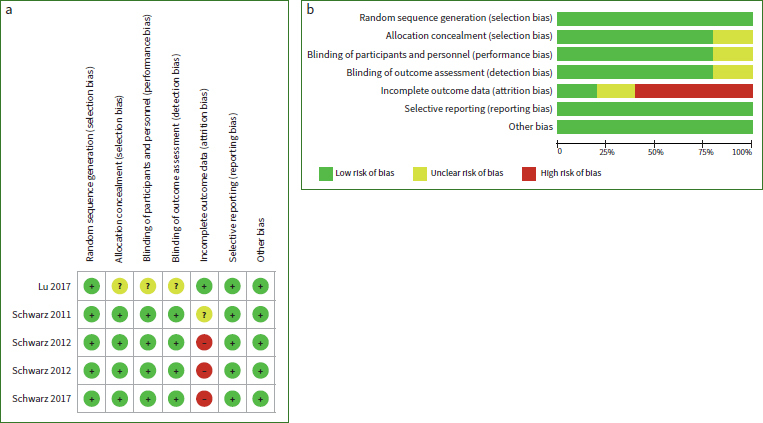
a) Risk of bias graph: review authors’ judgments about each risk of bias item for each included study; b) risk of bias summary: review authors’ judgments about each risk of bias item presented as percentages across all included studies.

The risk of bias and summary are presented graphically in Fig 2. Out of five studies, four^17,26,28,29^ were considered as high risk of bias, while one^27^ was considered as unclear risk of bias.

### Laser and Photosensitiser Parameters of Included Studies

As shown in Table 2, all included studies used ERL with wavelengths of 2940 nm. Energy fluence (J/cm^2^) was not reported in one study;^17^ in the other four studies, it was 11.4 J/cm^2^. Power output in one study^17^ was 1.2 W, and 1 W in the other four studies.^26-29^ None of the included studies reported the irradiation time or optic-fiber diameter.

#### Main outcomes of studies

##### CAL Gain

Only one study^17^ reported a statistically significant difference in terms of implant CAL gain (p < 0.05) in favour of the experimental group when compared with the control group. The other four studies^26-29^ did not find any statistically significant differences between the two groups (p > 0.05).

##### PD reduction

One study^17^ reported a statistically significant difference in terms of implant PD reduction (p < 0.05) in favour of the experimental group when compared with the control group. In contrast, one study^27^ did not detect statistically significant differences between the two groups (p > 0.05). With the extension of the follow-up period (up to 85 months), three more studies^26,28,29^ also failed to find a statistically significant difference between the two groups (p > 0.05).

##### PI reduction

None of the five studies^17,26-29^ determined a statistically significant difference between the two groups in terms of PI reduction(p > 0.05).

##### BOP change

One study^17^ did not report the outcome BOP. Four studies^26-29^ found no statistically significant difference between the experimental group and the control group in terms of BOP change (p > 0.05).

## Discussion

The present systematic included five clinical studies that reported on outcomes of Er:YAG laser therapy in the surgical treatment of advanced peri-implantitis. The selection criteria for collecting relevant studies only allowed the inclusion of randomised controlled clinical trials with an adequate number of patients and follow-up time to maintain a high level of evidence. Compared with baseline, the statistical analysis revealed that all included studies found improvement in the condition of peri-implantitis treated with Er:YAG laser combined with the surgical method. Such findings are consistent with a prospective consecutive case series evaluation,^2^ which revealed that a combination of Er:YAG laser decontamination and surgical debridement coupled with a regenerative approach could result in statistically significant pocket depth reduction and radiographic bone-fill of peri-implantitis bony defects. Regarding the comparison of the amelioration of peri-implantitis between the experimental group and the control group, the findings are discussed in the following.

In terms of the primary outcome CAL gain, one studiy^17^ revealed a higher increase in CAL in the experimental than in the control group. In contrast, the other four studies^26-29^ showed a higher increase in the control than in the experimental group. Among the studies, only one^17^ reported a statistically significant difference in terms of implant CAL gain (p < 0.05) in favour of the experimental group. The other studies^26-29^ did not find any statistically significant difference between the two groups (p > 0.05).

With regard to the secondary outcome PD reduction, two studies^17,26^ revealed a higher decrease in PD in the experimental than in the control group. The other three studies^27-29^ showed a greater decrease in the control than the experimental group. Only one study^17^ reported a statistically significant difference in terms of implant PD reduction (p < 0.05) in favour of the experimental group. The other studies^26-29^ failed to find statistically significant differences between the two groups (p > 0.05). For the secondary outcome full-mouth PI, all mean values were maintained at a low level. For the secondary outcome full-mouth BOP, four studies^26-29^ found no statistically significant difference between the experimental group and the control group (p > 0.05). One study^17^ did not report BOP data.

The treatment of peri-implantitis implies the decontamination of the surface of the implants. Although the process of roughening the surface of the implant can improve osseointegration, the exposure of threads can also cause an increase in bacterial adhesion. In the presence of deep pocket and bone defects, surgical access to peri-implantitis lesions simultaneously facilitates the removal of all granulation tissue from the defect area and a thorough debridement and decontamination of exposed implant surfaces. The aim of surgical intervention should be the thorough debridement and the repositioning of the marginal mucosa, in order to enable the patient to perform effective oral-hygiene practices.^6,7^

There are several means of debridement and decontamination of exposed implant surfaces, such as the conventional methods of mechanical debridement (ultrasonic cleaning with a carbon-fiber tip, sandblasting, plastic or titanium curettage) and antimicrobial treatment.^21^ Although conventional methods are effective in removing plaque, the threads are exposed after peri-implant inflammation occurs. Unlike natural teeth, which can be handled by smoothing the root surface through subgingival scraping, mechanical debridement methods causes damage to the threaded microstructure on the surface of the implant. Furthermore, because of the existence of threading, harmful substances such as foreign matter, bacteria and toxins are likely to remain in the thread, which renders thorough cleaning of the infected implant surface difficult and probably results in reinfection. As such, conventional methods are effective but have limitations.^31^

Due to its high affinity for water, the Er:YAG laser can emit light at a wavelength of 2940 nm, and thus can be applied on both soft and hard tissues. Considering that the reflection capacity of titanium for Er:YAG light is 71%, implant surfaces do not absorb the irradiation, and subsequently, the temperature does not increase during the decontamination processes. In this way, no damage should occur to the implant surface.^30^

Several in-vitro studies focused on the effect of Er:YAG laser on the implant surface. Scarano et al^25^ revealed that Er:YAG laser at different settings had varying degrees of impact on the surface of the implant: minor surface alterations caused an increase in superficial oxide level, as well as a decrease in porosity and microroughness, representing a positive alteration that could protect the materials against bacterial adhesion. Nejem et al^19^ concluded that a low-energy Er:YAG laser applied in three passages appeared to be an encouraging approach in decontamination of implant surfaces. In one recent study,^34^ the results suggested that Er:YAG laser irradiation at clinically relevant settings had no essential effect on osteogenic gene and protein expression of osteoblasts, and caution was advised for the clinical treatment of peri-implant diseases using Er:YAG laser.

Several clinical studies focused on the additive effect of Er:YAG laser on surgical treatment of peri-implantitis. A case report concluded that Er:YAG laser-assisted bone-regenerative therapy (Er-LBRT) could be useful and effective for the management of multiple peri-implant bone defects.^16^ Norton et al^20^ concluded that Er:YAG laser could be a useful adjunctive tool in the decontamination of implant surfaces. In combination with surgical regenerative therapy, Er:YAG laser can reduce the pocket depth by an average of 2.8 mm and possibly reduce tissue adherence with a mean defect fill of 1.43 mm.

Yan et al^35^ suggested that use of the Er:YAG laser as an alternative to subgingival mechanical debridement could potentially provide additional short-term benefits, while there was no evidence of superior long-term effectiveness. Lin et al^15^ found that laser therapy in combination with surgical/non-surgical therapy provided a minimal benefit in PD reduction, CAL gain, and PI reduction in the treatment of peri-implant diseases. Chala et al^1^ found that the adjunctive use of lasers in the treatment of peri-implant inflammation was effective for up to three months. However, strong evidence was not provided regarding the long-term benefits compared with conventional treatment. Overall, the conclusions of the aforementioned meta-analyses are similar to the conclusion of the present study.

In the evaluation of the systematic review design, the assessment of quality and risk of bias for all included studies was crucial. Several factors potentially influenced the heterogeneity, including various bone defect types and different non-surgical debridement methods. In addition, three studies^2^^6,^^28^^,^^29^ were considered as having a “high” risk of bias because some patients were lost to follow-up and the outcome data were incomplete.

Several potential limitations were identified in the present analysis. Four studies^26^^-^^29^ referred to the classifications of bone-defect type and described the results based on the classifications but did not present any statistical analysis. Further, the included studies were limited to English or Chinese, which may have increased the bias and resulted in relevant studies in other languages being missed. Finally, the number of the included studies was low.

## Conclusions

This systematic review demonstrated inconclusive findings on the efficacy of Er:YAG laser as a debridement method in surgical treatment of advanced peri-implantitis due to methodological heterogeneity such as non-standard control groups and laser parameters.The results of this review should be considered preliminary and further, well-designed studies with standardised comparators with laser parameters are warranted.
